# Supplementary material for: Comparative Study of Antimicrobial Activity of Silver, Gold, and Silver/Gold Bimetallic Nanoparticles Synthesized by Green Approach
Source: Molecules. 2022 Nov 15;27(22):7895. doi: 10.3390/molecules27227895 (PMC9694914; doi:10.3390/molecules27227895)
Supplement: Supplementary file 1 [file molecules-27-07895-s001.zip › molecules-2015213-supplementary.pdf]

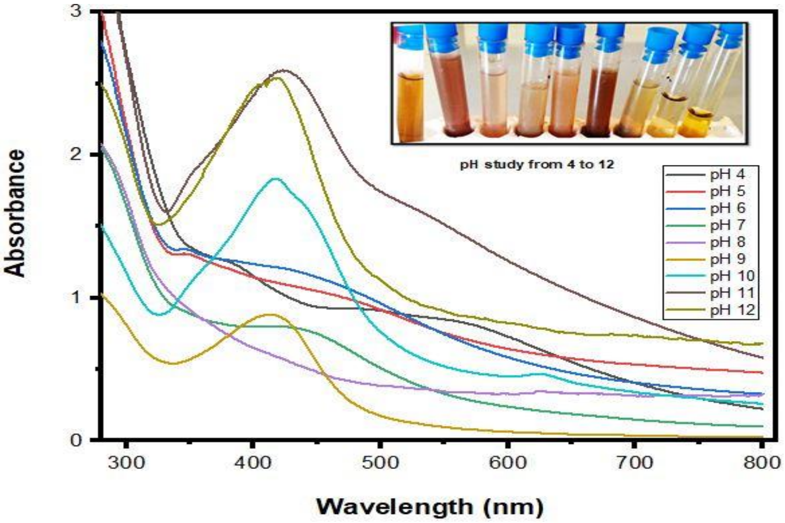
pH study from 4 to 12
pH 4
pH 5
pH 6
pH 7
pH 8
pH 9
pH 10
pH 11
pH 12
Absorbance
Wavelength (nm)

A

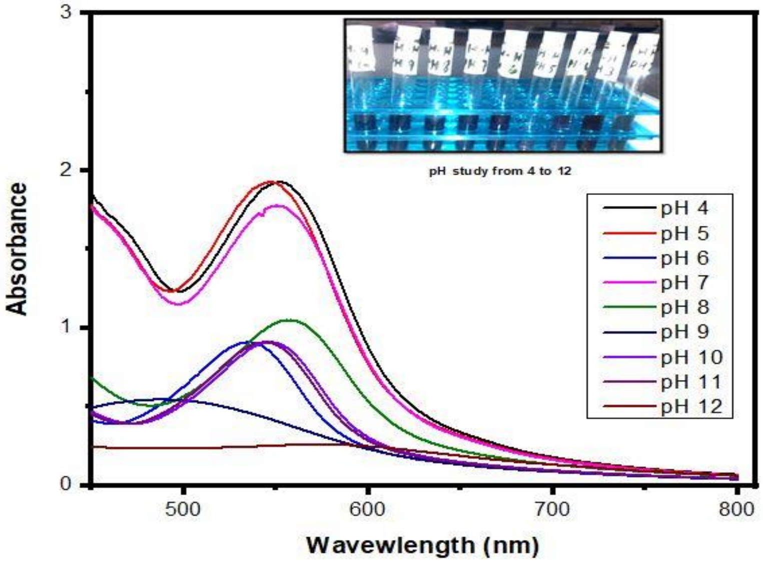
pH study from 4 to 12
pH 4
pH 5
pH 6
pH 7
pH 8
pH 9
pH 10
pH 11
pH 12
Absorbance
Wavewlength (nm)

B

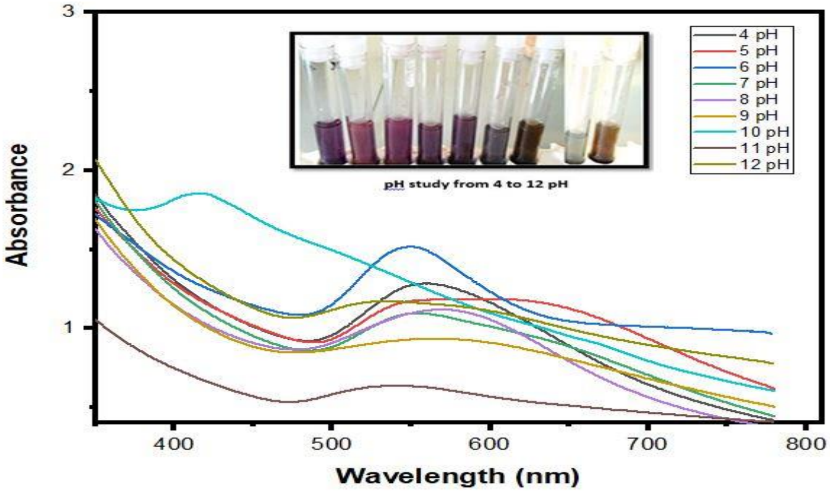
pH study from 4 to 12 pH
4 pH
5 pH
6 pH
7 pH
8 pH
9 pH
10 pH
11 pH
12 pH
Absorbance
Wavelength (nm)

C

Figure S1. UV-vis spectrum of NPs biologically synthesized by using the HH plant extract at different pH (Both acidic and basic). (A) HH-AgNPs, (B) HH-AuNPs, and (C) Ag/Au BNPs.

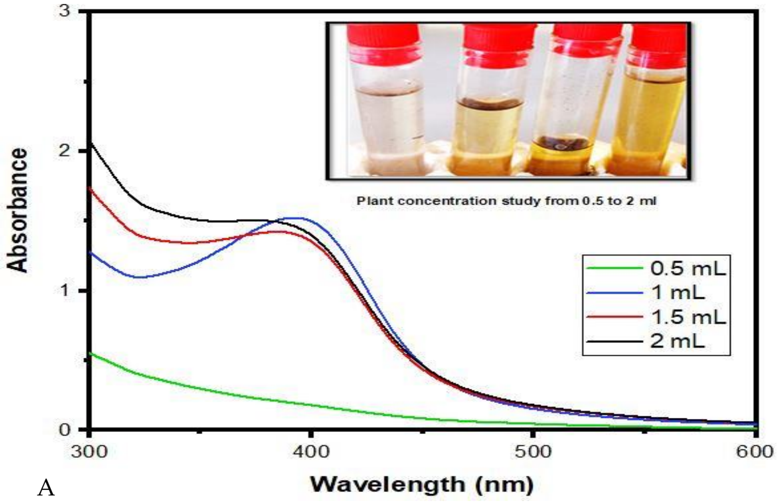

A

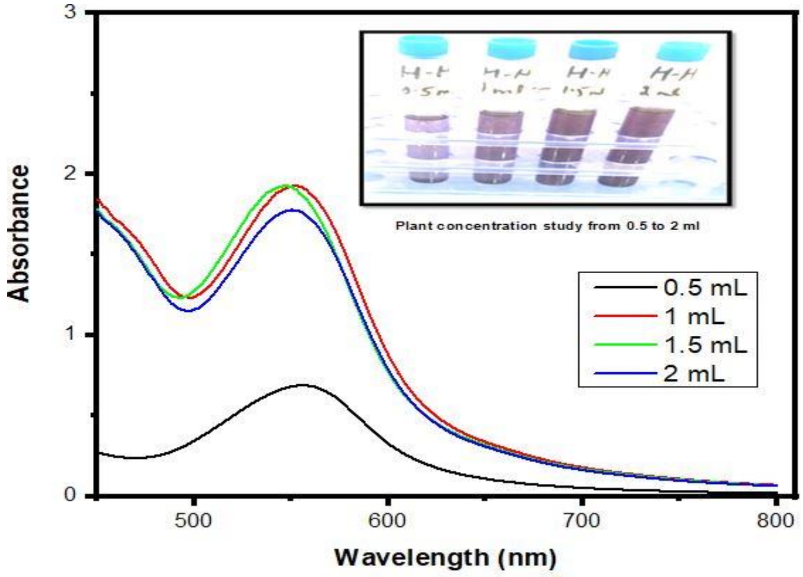

B

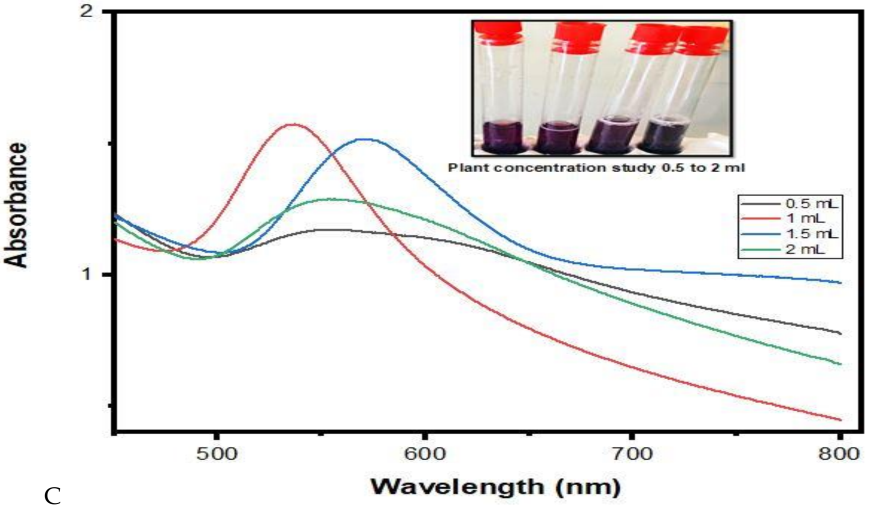

C

Figure S2. UV-vis spectrum of biologically synthesized NPs by using the different concentrations of HH plant extract. (A) HH-AgNPs, (B) HH-AuNPs, and (C) Ag/Au BNPs.

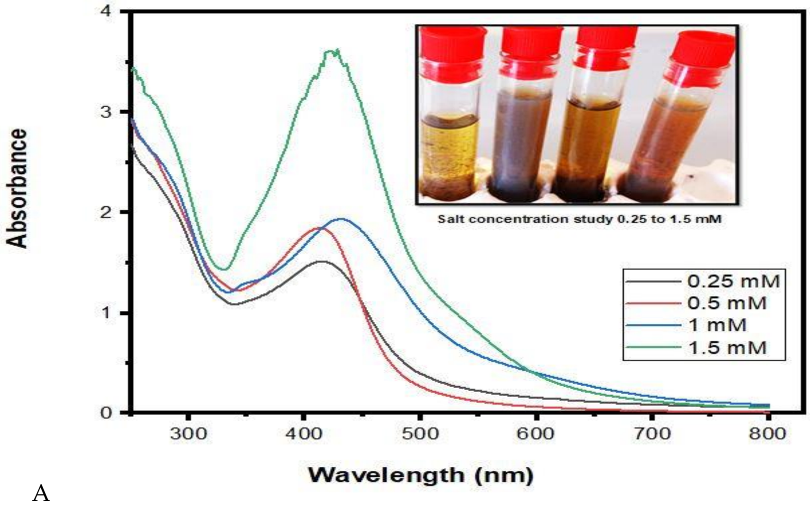

A

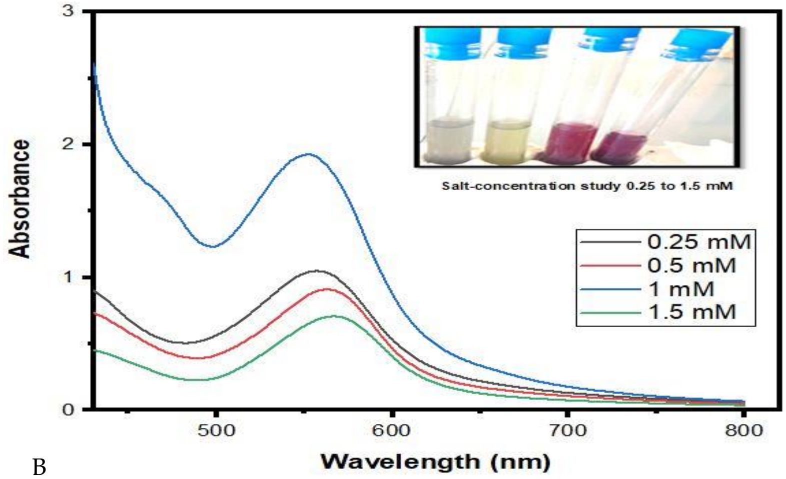

B

B

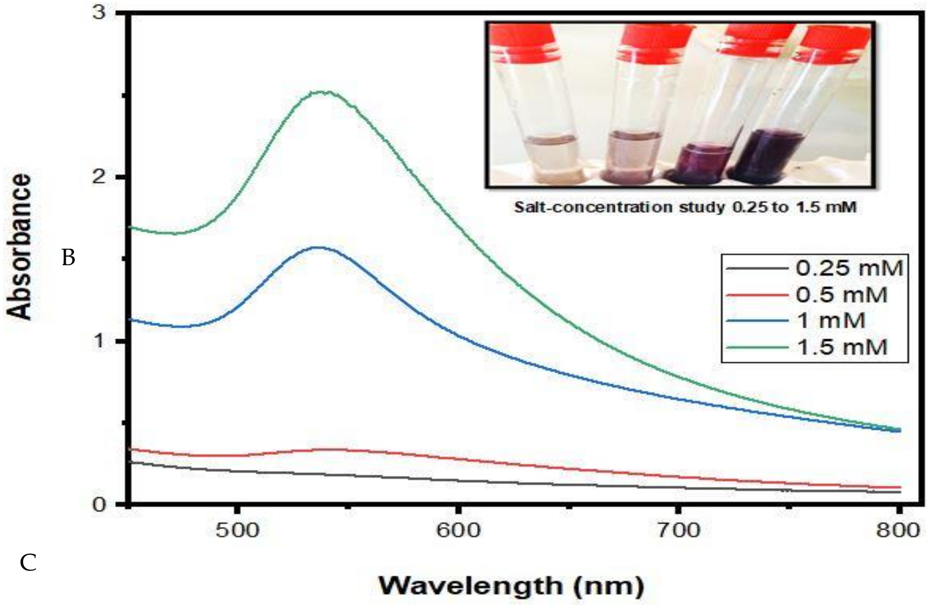

C

Figure S3. UV-vis spectrum of biologically synthesized NPs by using the HH plant extract at different concentrations of $AgNO_3$/$HAuCl_4.3H_2O$ salts. (A) HH-AgNPs, (B) HH-AuNPs, and (C) Ag/Au BNPs.

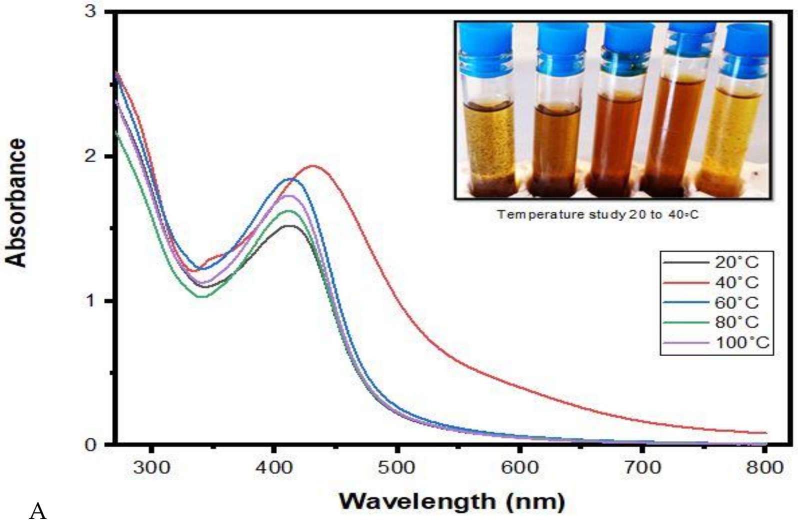

A

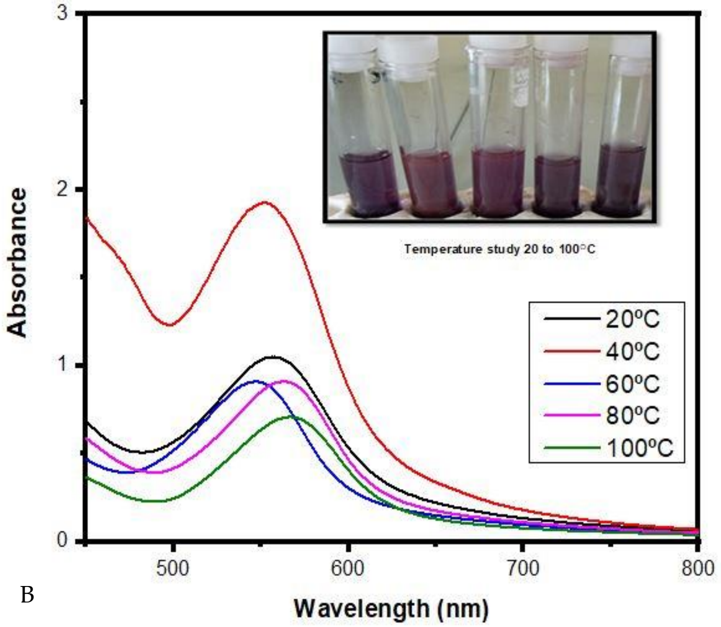

B

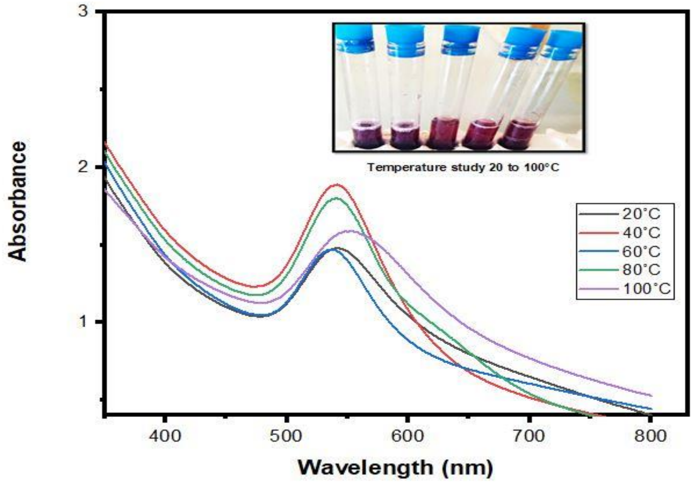

C

Figure S4. UV-vis spectrum of biologically synthesized NPs by using the HH plant extract at different temperatures. (A) HH-AgNPs, (B) HH-AuNPs, and (C) Ag/Au BNPs.

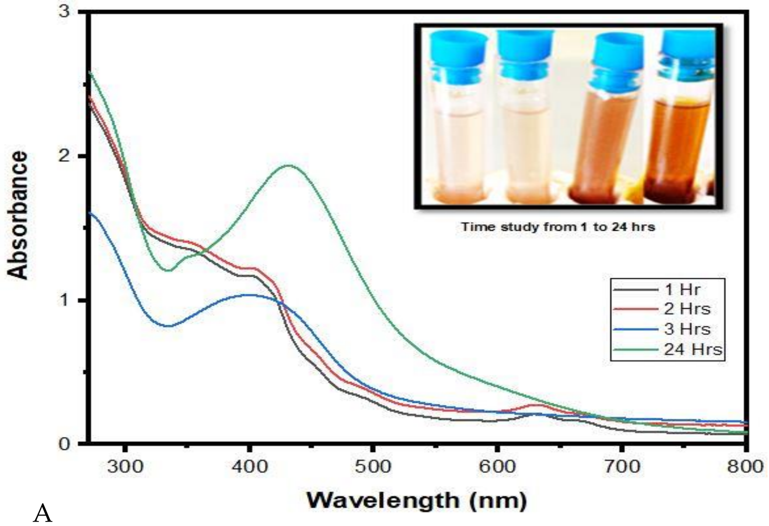

A

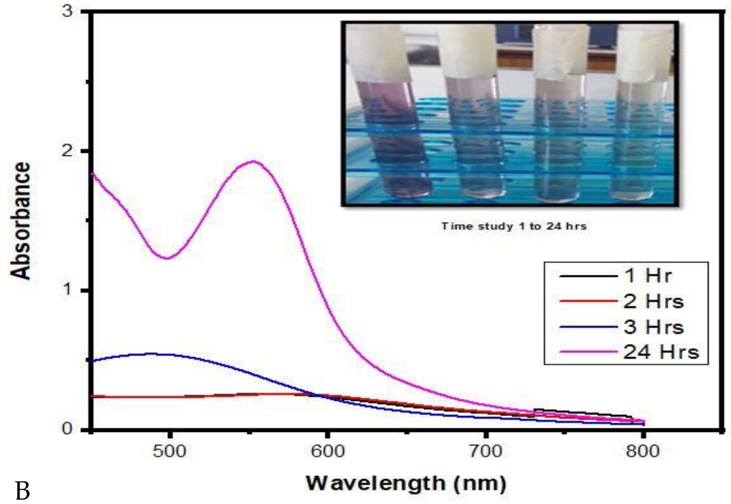

B

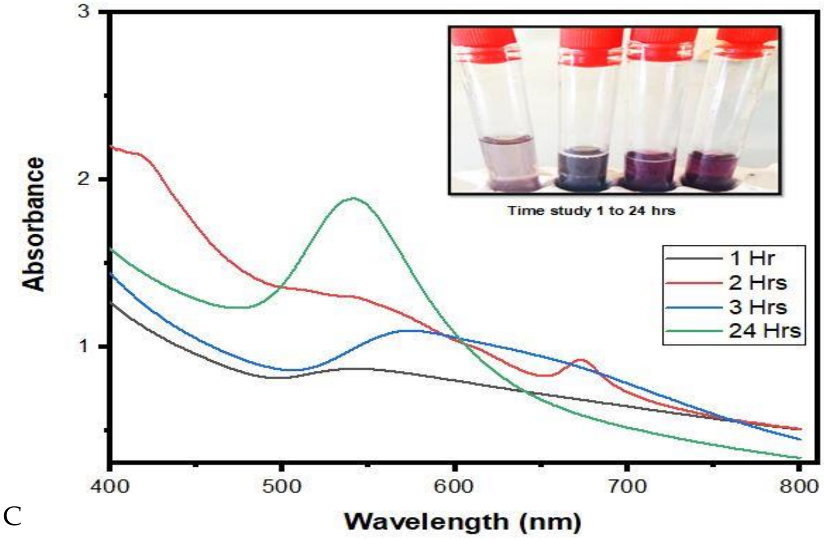

C

Figure S5. V-vis spectrum of biologically synthesized NPs by using the HH plant extract at different reaction times A) HH-AgNPs, (B) HH-AuNPs, and (C) Ag/Au BNPs.

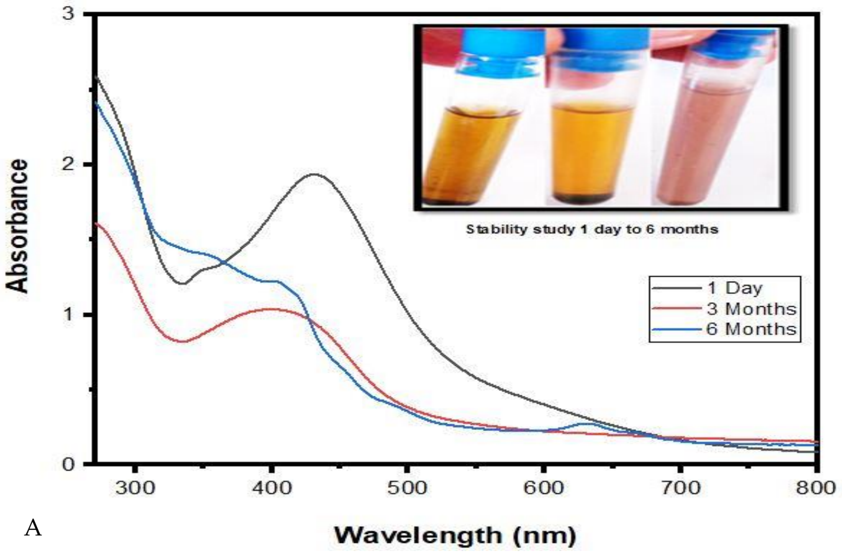

A

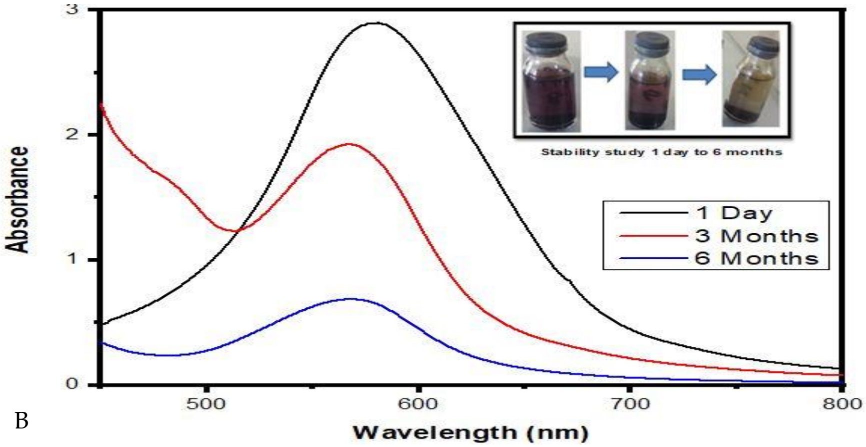

B

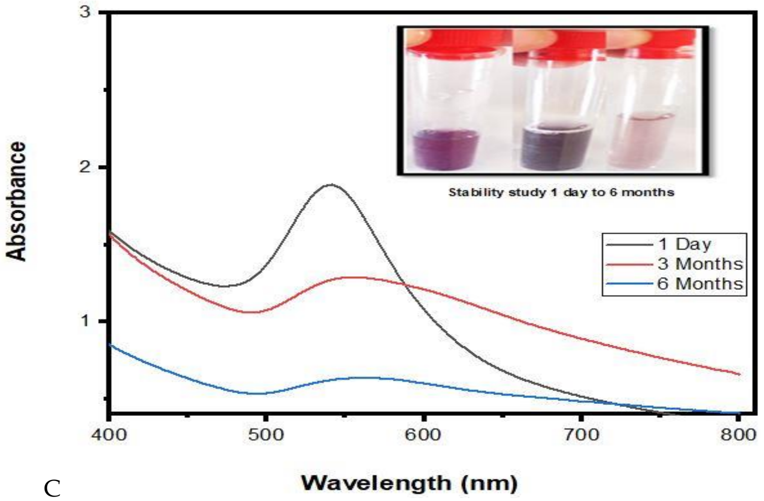

C

Figure S6. V-vis spectrum of biologically synthesized NPs by using the HH plant extract at different periods. (A) HH-AgNPs, (B) HH-AuNPs, and (C) Ag/Au BNPs.

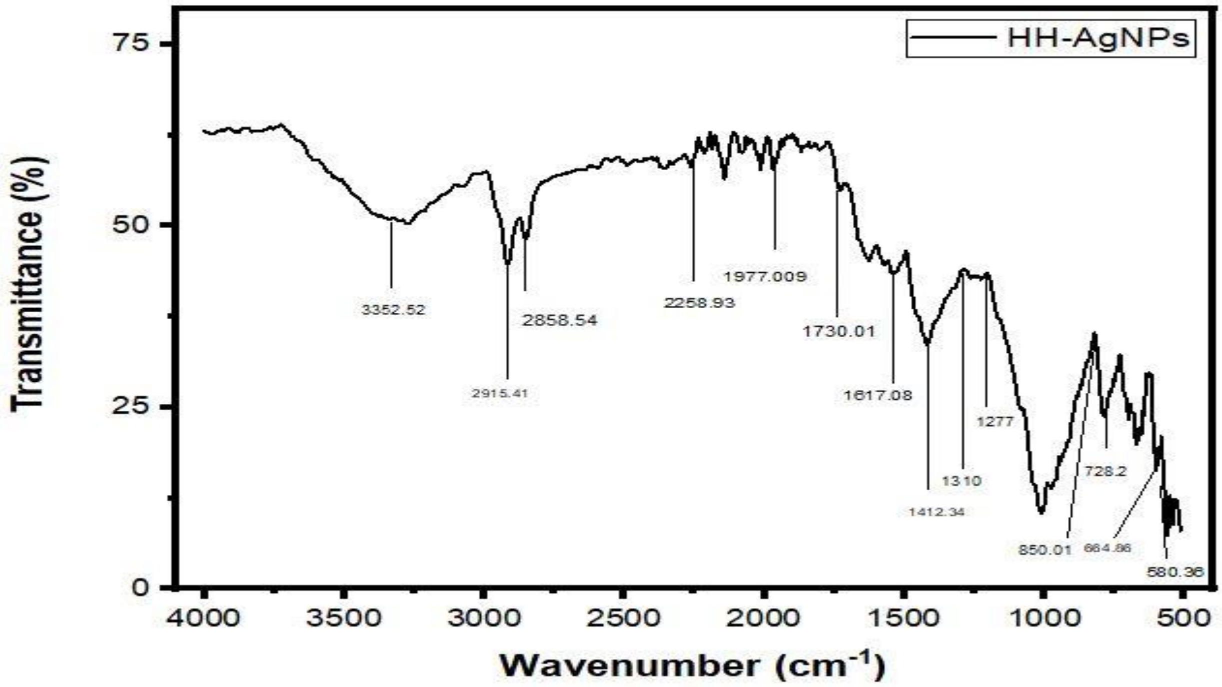

Figure S7. FT-IR analysis of HH-AgNPs.

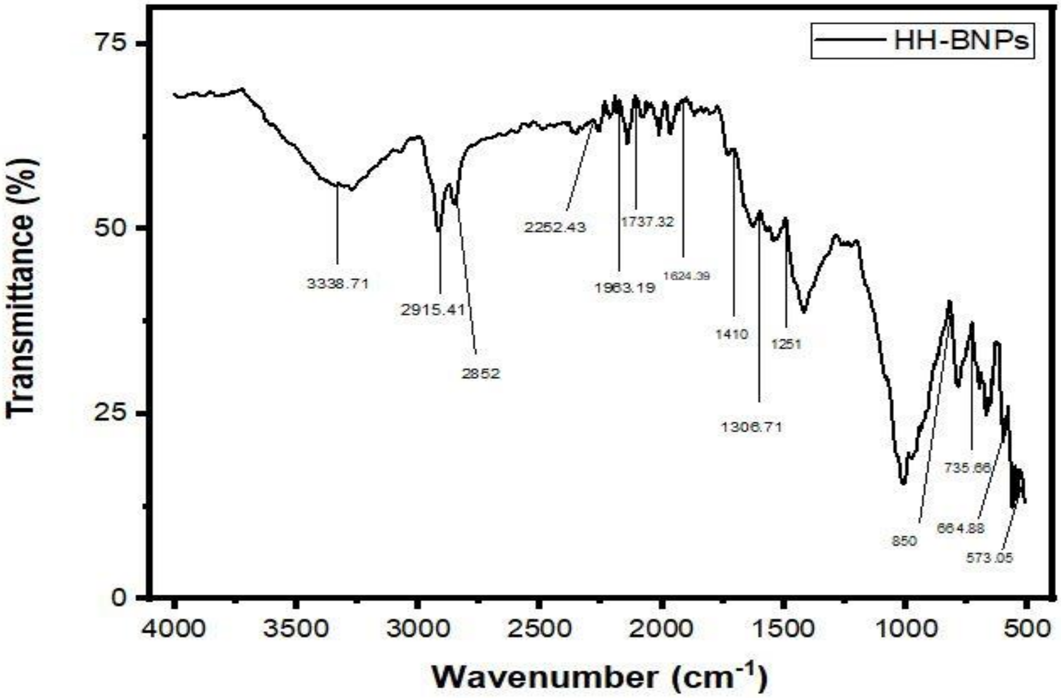

Figure S8. FT-IR analysis of HH-BNPs.

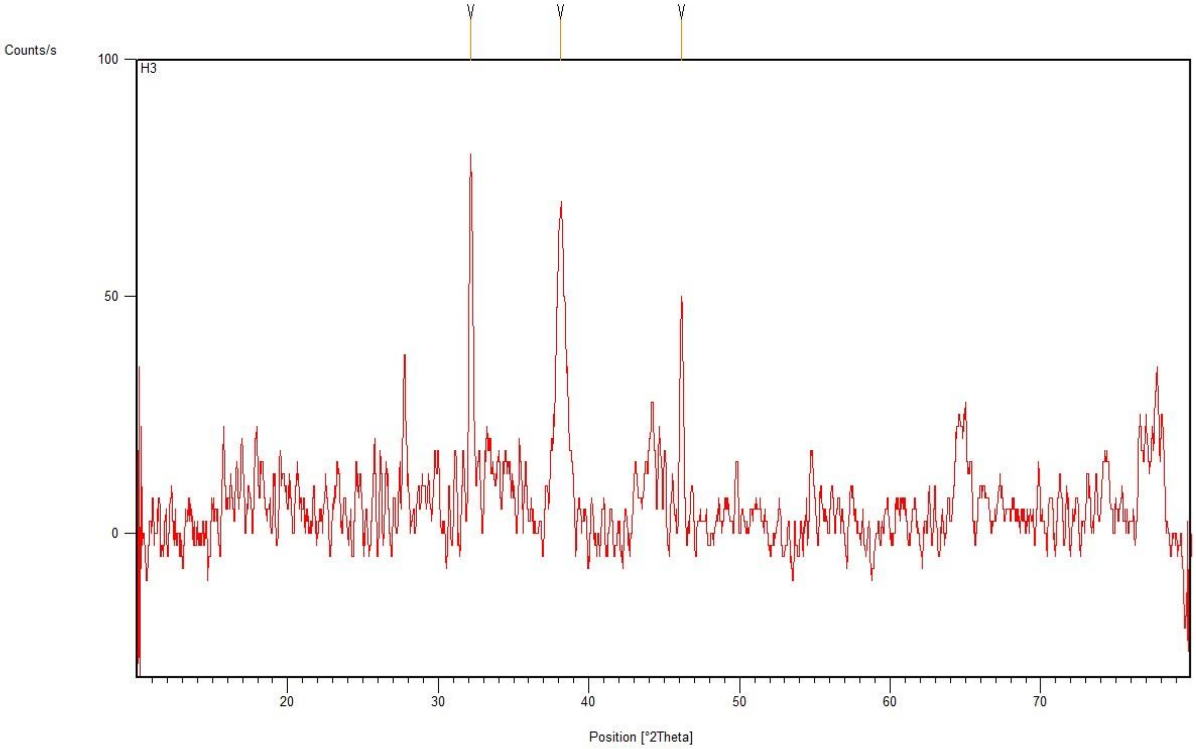

Figure S9. XRD analysis of HH-BNPs.

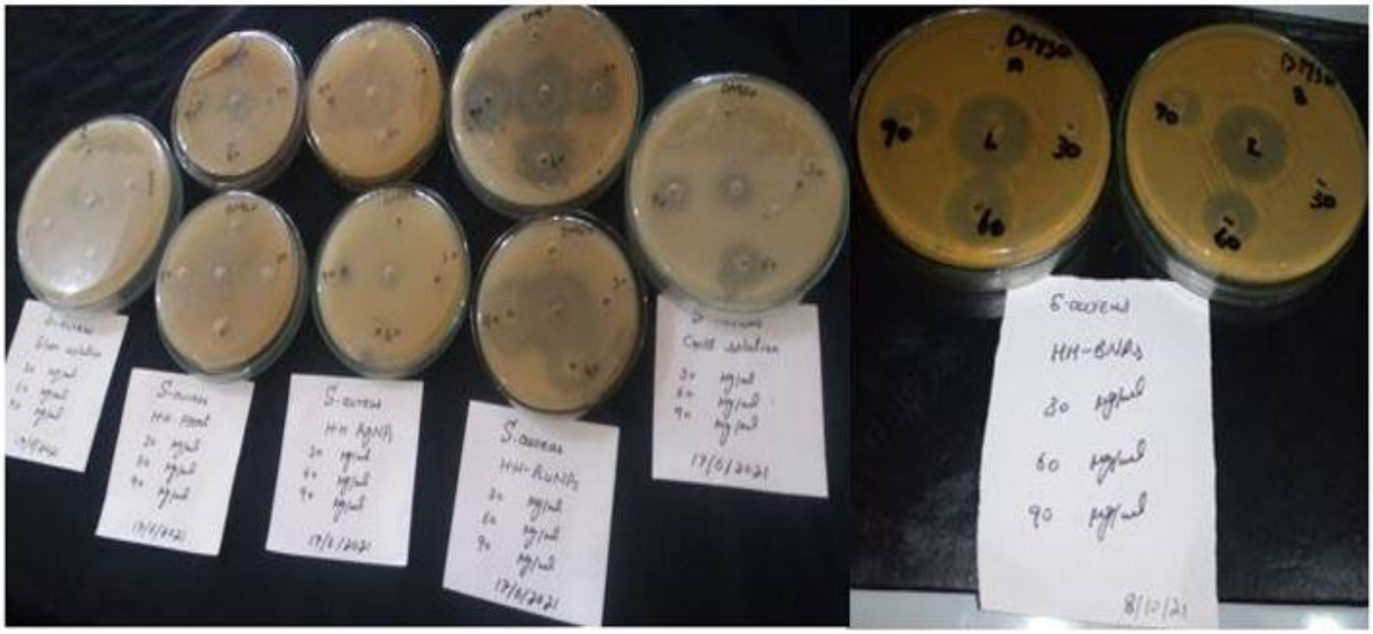

***S. aureus* HH-samples**

A

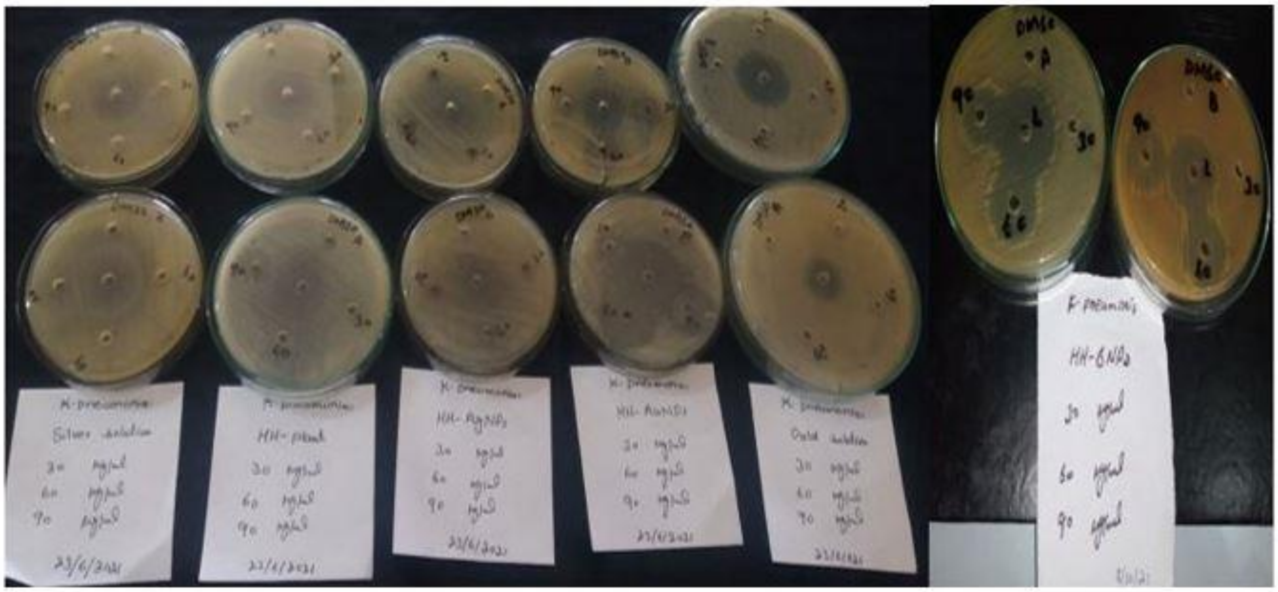

***K. pneumonia* HH-samples**

B

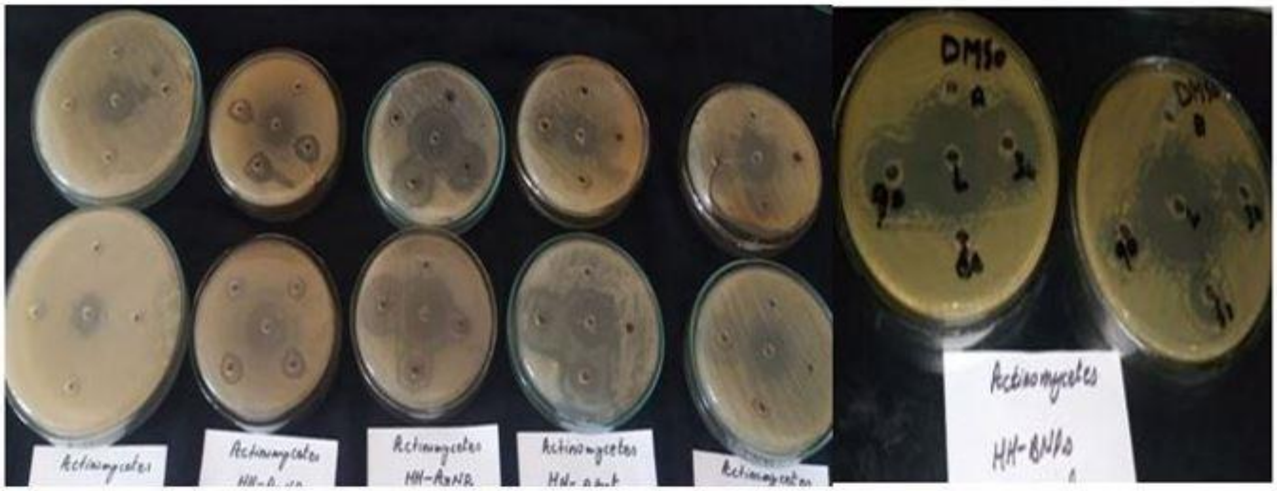

***A. meriye* HH-samples**

C

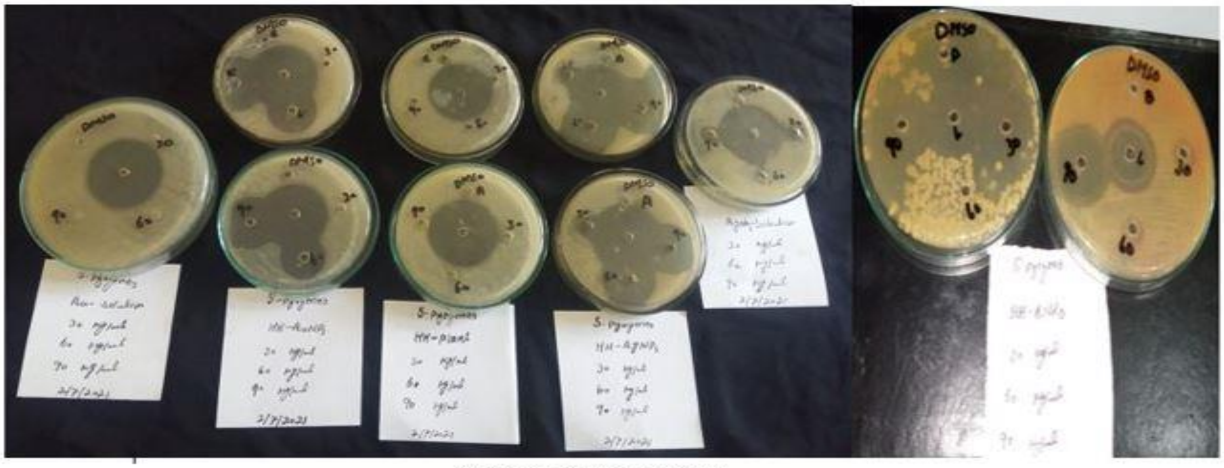

***S. pyogen* HH-samples**

D

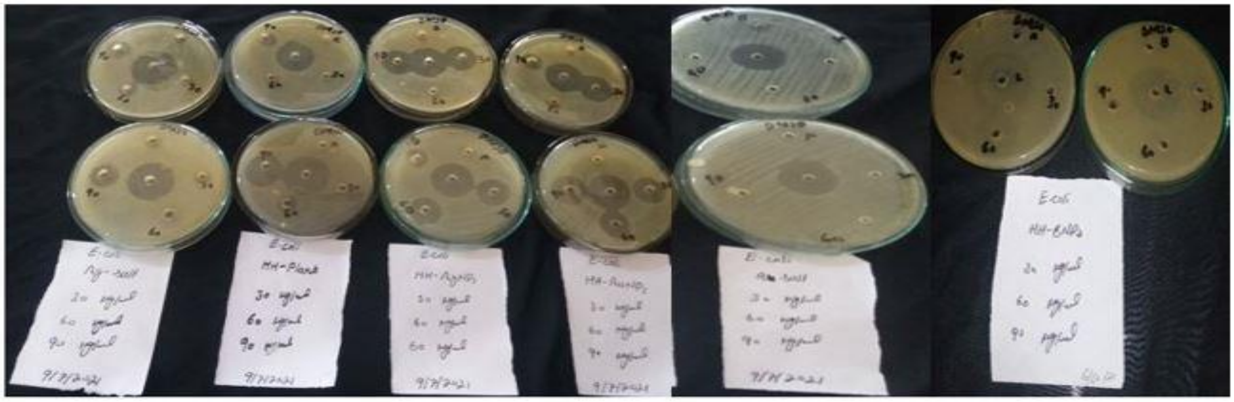

***E. coli* HH-samples**

E

E

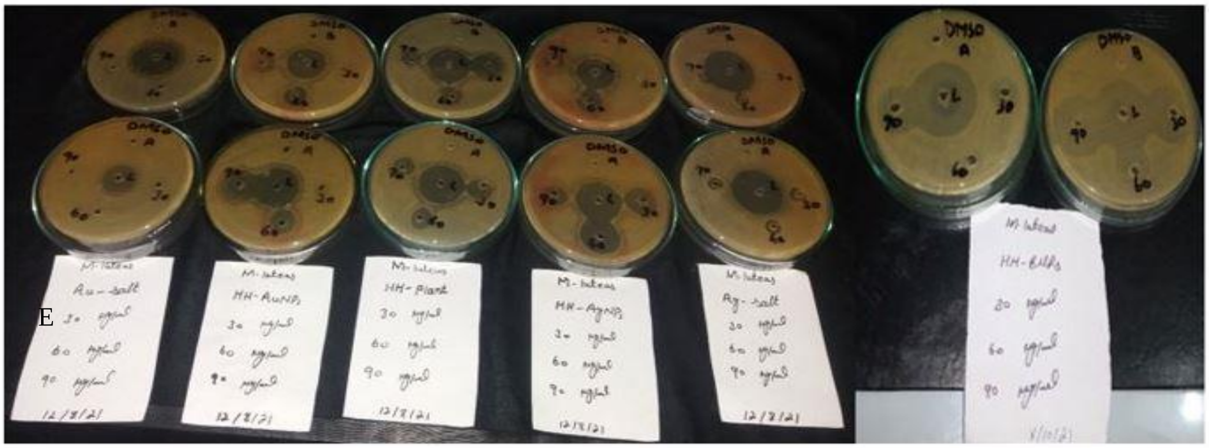

S. marcescens **HH-samples**

F

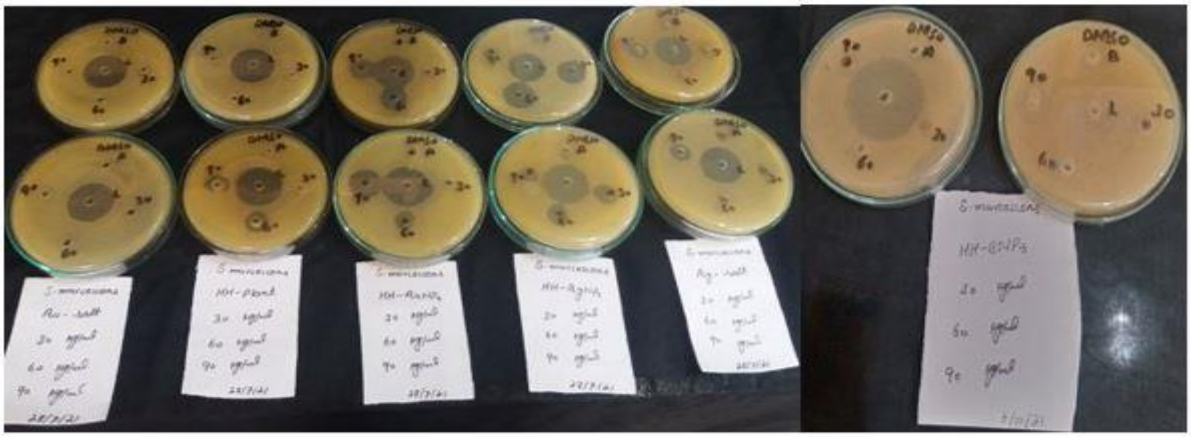

B. cereus **HH-samples**

G

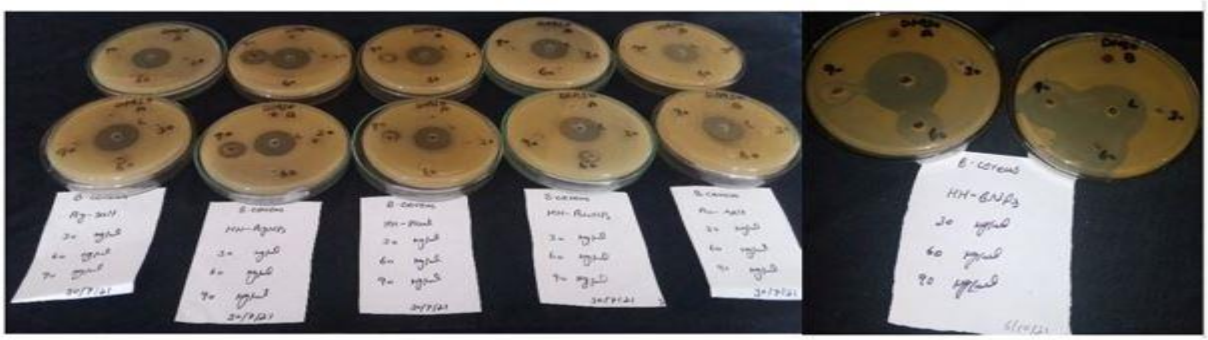

MRSA **HH-samples**

H

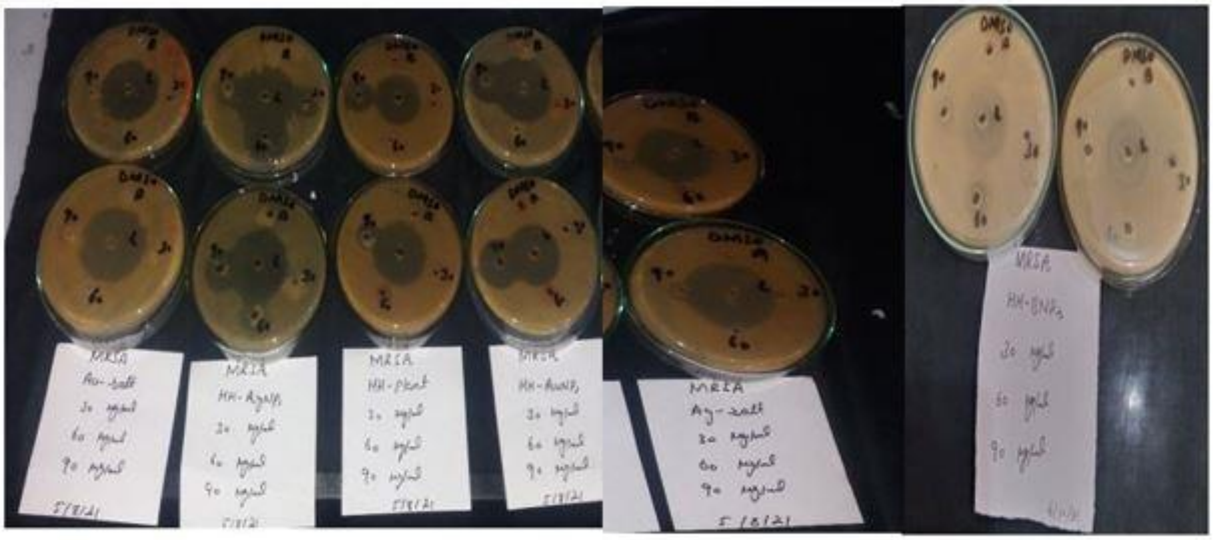

***M. luteus* HH-samples**

I

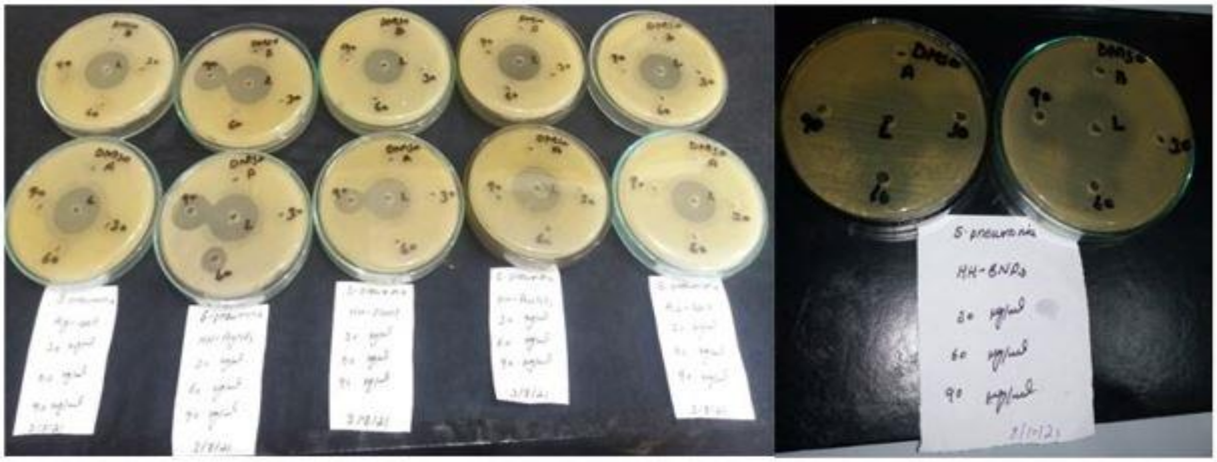

***S. pneumonia* HH-samples**

J

Figure S10. (A) S. aureus zone of inhibition (B) K. pneumonia zone of inhibition (C) A. meriye of inhibition (D) S. pyogenes zone of inhibition (E) E. coli zone of inhibition (F) M. luteus zone of inhibition (G) S. marcescens zone of inhibition (H) B. cereus zone of inhibition (I) MRSA zone of inhibition (J) S. pneumonia zone of inhibition (J) zone of inhibition.

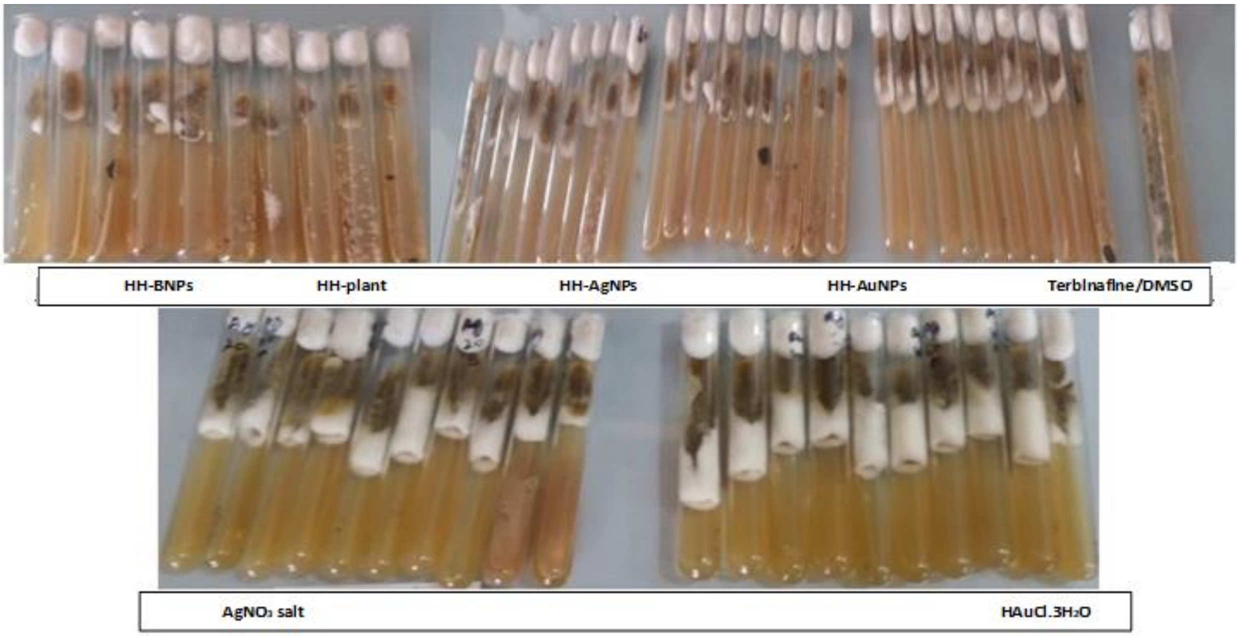
HH-BNPs
HH-plant
HH-AgNPs
HH-AuNPs
Terblnafine/DMSO
$AgNO_3$ salt
$HAuCl.3H_2O$
A

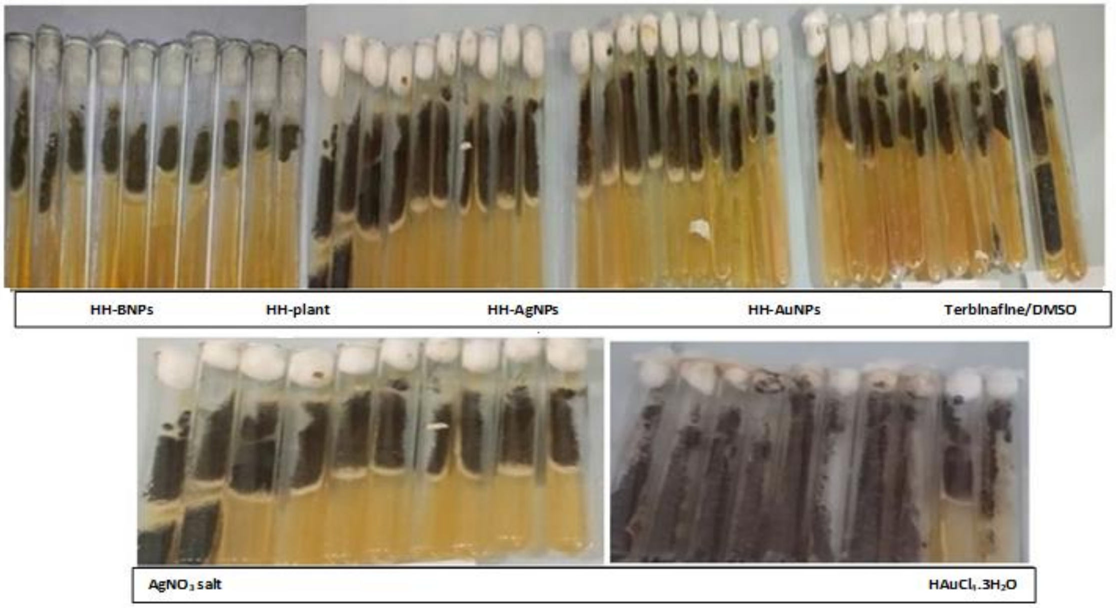
HH-BNPs
HH-plant
HH-AgNPs
HH-AuNPs
Terblnafine/DMSO
$AgNO_3$ salt
$HAuCl_4.3H_2O$
B

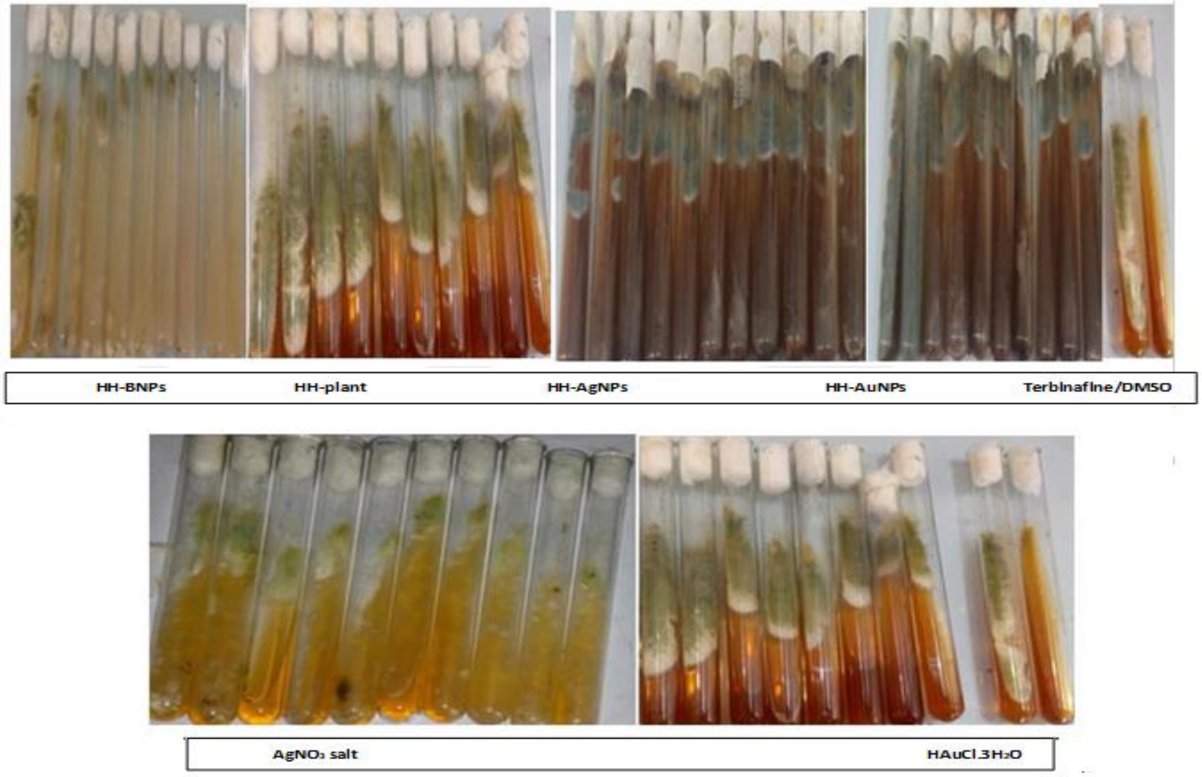

C

Figure S11. (A) *A. niger* growth inhibition, (B) *A. fumigatus* growth inhibition, and (C) *A. flavus* growth inhibition.
